# Cases of Hydrophobia, with the Appearances on Dissection, and Observations on the Means of Prevention

**Published:** 1807-04

**Authors:** M. Sabatier

**Affiliations:** Surgeon to the Hotel des Invalids, at Paris


*Cases of Hydrophobia, with the Appearances on Dissection, and Observations on the Means of Prevention*; by M. SABATIER, Surgeon to the Hotel des Invalids, at Paris.

( Concluded from our last, p. 218—225. )

HE was much agitated, and threw his body into various contortions, which the bye-standers regarded as proofs of insanity. The physician, who saw him at four o'clock in the afternoon, ordered him to be bled in the foot. The patient, at this time, experienced great horror at the sight of water; and began to squirt out saliva to a considerable distance. He believed it to be the intention of the attendants to smother him beneath the bed-clothes; and it was not without much difficulty that they could convince him this opinion was erroneous. At six o'clock, when I arrived, I found him somewhat more calm; nevertheless, he uttered frequent groans, so much resembling that of the former patient, that my domestic, whose curiosity had induced him to follow me, hearing him at at a distance, believed him to be affected with the same malady.

The unfortunate man knew me very well, and earnestly inquired, if I thought it impossible to save him. The greatest distress he experienced, proceeded from the incapability of swallowing fluids, and from violent spasms in his lower jaw and throat. He constantly threw out saliva; and his features were so much distorted as to be scarcely recognizable. He continued to groan; his pulse was small and hard, and his skin cold. I asked him if he was able to drink, on which he expressed his readiness to make the attempt, and taking a pewter vessel in his hand containing some liquid, he regarded it a long time with great horror, and without daring to carry it to his mouth; he at last hastily seized it between his teeth, and poured a few drops drops of the liquid into his mouth, which, however, he found it impossible to swallow, and instantly rejected it, while his whole frame experienced the most violent convulsions. A second attempt was not more successful; on the third, however, he succeeded in getting down, at a single gulp, the small quantity of liquid he had taken into his mouth; but this was not accomplished without the greatest effort, and was attended with the same convulsive agitations as on the first trial. I then withdrew, thoroughly convinced that he was become hydrophobic, and that his situation was in consequence of the wounds he had received from the dog.

As I foresaw that he would most probably die that night, or the following day, I prescribed no medicine, but left orders to confine him if necessary, in order to prevent any accident, in case he should become furious. He remained, however, more tranquil after my last visit; though he still occasionally experienced considerable agitation, and at other times complained more than usual. Death terminated his sufferings about half past twelve at night.

The next day, 28th, I proceeded to the examination of the body. The pharynx, œsophagus, and stomach, having been laid open through their whole extent, I found the *velum pendulum palati* somewhat red, and appearing as if slightly inflamed; about a spoonful of greyish coloured mucous matter was collected from the superior part of the pharynx, and its inferior part, opposite the lower edge of the thyroid cartilage, was contracted for about two fingers breadth, so as scarcely to admit the introduction of a common quill.

The stomach, without being sensibly contracted, displayed a greater degree of rugosity than usual; all the other viscera appeared in a natural state.

In consequence of the termination of this case, I could not wholly divest myself of anxiety respecting the fate of the subaltern officer, who had been injured by the same animal. It was true, the wound which he received, was inflicted through his shoulder-belt and clothes, and might therefore be regarded as comparatively harmless, since the teeth of the animal must have been by this means in a considerable degree freed from the virus before reaching the skin; it is however certain, that hydrophobia has been known to occur from wounds so slight, that little or no apprehension was entertained for the consequences. What however rendered me more confident of his safety was, that the wound had been twice cauterized, and the suppuration kept up for a considerable length of time. I saw this patient after such a long interval of time, as fully warrants me to state, that he had either received no infection, or that the effects usually resulting from it had been prevented by the mode of treatment which was adopted.

The cases, which remain to be noticed, do not exhibit such melancholy results as the foregoing, since all the patients escaped without being affected by the disease.

On a careful re-perusal of the original notes from which these cases were drawn, I am fully convinced however, that the animals, by which the patients were injured, had actually been in a rabid state, and consequently that it was either owing to the preventive means employed, that the disease did not follow, or to a want of susceptibility in the patients at the time, for in rabid hydrophobia, as in all other contagious maladies, if the necessary predisposition does not concur, no disease will follow from the application of the virus.

On Thursday, the 21st of October, 1780, an officer of Invalids perceived that his dog seemed disinclined to take any supper; and on the Friday and Saturday following, the animal refused all kinds of food whatever. When following his master in the *Boulevards,* he attacked and wounded three large dogs; and on the day following (Sunday), three other dogs and a cat were bitten by him. His eyes were haggard, he ran to and fro, evincing a great disposition to attack every animal that fell in his way, but did not offer to injure those persons who happened to approach him; nor is it in the least probable that he would have bitten his master, had he not endeavoured to force him to swallow some theriaca, which had been recommended as a remedy for the malady.

The officer was wounded in the last phalanx of the fore finger of the left hand, and the subaltern officer who assisted to hold the animal was bitten in the thumb of the right hand. His patience being at length exhausted by the obstinacy of the dog in refusing to swallow the potion, he seized him by the hind feet, and dashed him on the pavement.

On Monday the 25th, some doubts having arisen respecting the true nature of the malady under which the dog had laboured, the patients were persuaded to apply for advice at the Hotel des Invalides, where they arrived about three o'clock in the afternoon. I saw them immediately; they were perfectly easy respecting their situation, and did not believe it necessary to submit to any preventive ventive treatment whatever. The circumstances already related rendered it, in my opinion, however, highly necessary; but it was with much difficulty, that the physician and I could make them comprehend, that in order to prevent the consequences which might possibly result from the injury they had sustained, it was necessary to amputate the phalanges of the fingers. On this day, however, they refused to submit to the operation; but a cathartic was prescribed in order to prepare them for it, should their consent be obtained on the following morning. Our opinion at last produced the desired effect, and on Friday the 27th, I amputated the last phalanx of the injured finger in each of the patients, about seventy-two hours after the accident. They were subjected to a proper regimen, for a short time, after which they rejoined their regiment, and have experienced no accident since.

The practice which I followed in this case, appeared to me best calculated to answer the proposed end, by preventing the absorption of the virus into the system; for it was scarcely possible sufficiently to cauterize the extremity of the thumb and fore-finger, since the skin of these parts is too hard to admit the fire to penetrate to a sufficient depth to ensure complete safety. Besides, serious inflammation might have followed from the practice; whereas, on the contrary, no disagreeable consequences could result from amputation, while at the same time, it afforded the patient complete safety. The patients themselves appeared to be very grateful for the attention I had bestowed on them, and especially for the pains I had taken to inspire them with sufficient courage to submit to the operation. Since that time, I have been informed, however, that they have entertained some doubts respecting the necessity of the operation. But admitting there might be even some cause of doubt, what reasonable man would not rather submit to a slight evil, than run the risk of being attacked by such a dreadful malady as hydrophobia, and one that has never yet been cured by any internal remedies whatever?

I was consulted, about the same period, by one of my friends, respecting a child, 8 years of age, who had been bitten by a dog in the fore-arm. Some suspicions were entertained respecting the state of this animal, which had unfortunately been too hastily killed. It is, however, certain, that another person bitten by the same dog, died under very suspicious circumstances, in the Hospice de l'Humanite at Paris. The child's wound bled profusely, and became covered with a sort of scab, or crust. Muriate of

 antimony antimony not being at that time employed as a caustic, I advised them to cauterize the injured part by means of burning tinder. An eschar about the size of a shilling was produced by this process, and after separating, the suppuration was kept up by an ointment, of which the red oxide of mercury formed a component part; as a means of farther security, or rather to tranquillize the relations of the child, mercurial frictions and the warm bath were employed at the same time. By the friend who consulted me respecting this case, I was informed a long time afterwards, that no disease had ensued.

The last patient bitten by a mad animal, for whom my advice was solicited, was the son of a magistrate, justly celebrated for his humanity. This young gentleman was wounded in several places in one leg; and all the circumstances attending the accident, concurred to warrant the most serious apprehensions respecting its consequences. On his parents applying to me, I advised the immediate cauterization of the injured parts; but it was with much difficulty, and not until after being informed of the state of the dog, previous to its death, and consulting several eminent practitioners, respecting the effect likely to result from the operation, that they would consent to have it put in practice. In this case, I employed the muriate of antimony, having first enlarged those wounds which were not sufficiently large to admit of the introduction of a dossil of lint charged with the caustic. The usual consequences resulted from this operation; it produced considerable pain and inflammation, which were followed by a copious suppuration, and the separation of the eschars. At the desire of his friends, mercurial frictions were employed, for though, in my opinion, they are wholly inefficacious, yet I did not wish to oppose a remedy which had been so highly extolled. The favourers of this practice will doubtless attribute the exemption of the patient from the disease to its use, while in my opinion, admitting that the dog was really in a rabid state, his security was wholly owing to the cauterization of the injured parts.

Including the case of 1784, it will be seen, that I have personally attended, or given advice to eleven patients bitten by dogs, some of which were ascertained to be mad, and others of them strongly suspected of being in that state. Out of these eleven, five died hydrophobic, three were preserved from the consequences of the infection, and the three others experienced no injurious effects from the accident, though it was extremely probable, from from all the circumstances taken in conjunction, that the animals by which they were injured, were at the time in a rabid state.

In those who died, the disease appeared at different periods from the time of the injury. The first patient was occasionally attacked with vertigo, from the very day on which he received the wound; in a short time, he complained of a weight in his head, and his ideas became confused. His features were much distorted; he passed restless and perturbed nights, and his sleep was continually interrupted by frightful dreams. Every symptom indicated approaching disease; but it did not assume a decided character, until the thirty-seventh day after receiving the injury. At this period, the convulsive agitation he experienced at the sight of water, removed every doubt respecting the true nature of his malady. In the second patient, a much greater interval elapsed between the infliction of the bite and the accession of the disease, than in the former case. The accident occurred on the 24th of December, and no symptoms of disease appeared until the 19th of the following March, eighty-six days after he had sustained the injury. The nature and situation of the wounds were, however, nearly the same; both patients having been bitten in the face; and it is an opinion extremely prevalent, that wounds in the face and head are more dangerous, than when inflicted on the extremities of the body.

The third patient was placed in still more unfavourable circumstances, having not only been injured in different parts of the body, but likewise in the crown of the head; besides, one of the wounds was much lacerated. In this case, the disease appeared at the end of fifty-two days.

The two last patients not having been under my own immediate care, I cannot speak with certainty as to the period at which the disease supervened, nor give a particular account of the symptoms by which it was characterized, or the precise time which elapsed between the first attack and the death of the sufferers. Among those whom I myself attended, the symptoms were nearly similar; convulsive spasms in the throat and neck, difficulty of swallowing, and extreme horror at the sight of liquids, dyspnœa, small and feeble pulse, distortion of the countenance, and extreme terror of death. They uttered low and deep groans, similar to the howling of a dog labouring under great pain; and a short time previous to death, a total loss of recollection ensued. Solids were swallowed,

 however, however, with considerable ease, which was so far favourable, as it admitted of the administration of medicines.—Neither did they wholly refuse liquids; for, however great might be their horror at the sight of water, and other fluids, they nevertheless endeavoured as far as possible to follow the advice which was given them, to swallow a few drops, and made the most strenuous efforts for that purpose, which were not in every instance unsuccessful.

During the continuance of their illness, I always approached these patients with the fullest confidence, since they never betrayed the least sign of being furious, or attempted to bite even during their delirious paroxysms. The only precaution which I employed, was to guard myself from the saliva, which, as has been mentioned, they squirted to a considerable distance.

It may be here proper to remark, that contrary to the generally received opinion, the wounds in none of the above instances either became painful, or the surrounding parts tumefied on the approach or during the continuance of the malady.

The duration of the disease was not the same in each of the patients. The first, though evidently affected from the beginning, if we are to judge from the presence of vertigo, &c. did not experience any decided symptoms of hydrophobia till five days previous to his dissolution, so that we can only reckon the duration of his malady at a hundred and eight hours. The second patient, who was attacked at a more distant period from the time of the accident, died at the termination of sixty hours from the first accession of the complaint, twenty-four of which were passed in tolerable ease and tranquillity, so that, in this case, the hydrophobic symptoms may justly be said to have been present only thirty-six hours.

In the third, the disease was characterized at once, the patient not having the slightest indication of its approach; it nevertheless terminated in death, at the expiration of forty-two hours.

On the inspection of the bodies, nothing preternatural appeared in those parts which, during life, seemed to be the peculiar seat of the disease. In the first patient, the mouth, pharynx, and œsophagus were in a natural state. In the second, the superior part of the œsophagus was whitish, and somewhat rugose, but did not exhibit any signs of inflammation. In the third, the palate appeared as if it had been inflamed, and the inferior portion of the pharynx was contracted for about two fingers breadth in extent.

extent. All the other parts of the body, and especially the brain and the thoracic and abdominal viscera, were in a sound state. In the two last cases, mercurial friction had been employed, and though the quantity rubbed in was not very great, yet it was certainly sufficient to have prevented the disease, had it possessed the efficacy attributed to it by many distinguished members of the profession. Another patient, it will be recollected, was put on a course of volatile alkali, but the result proved equally inauspicious.

It is not my intention to deduce any positive conclusion from these facts, my only design, in mentioning them, is to assist practitioners, who may be called upon, in the exercise of their profession, to attend patients under similar circumstances, to form a just estimate of the value of these two remedies, and not to rely too implicitly on their so much boasted prophylactic powers.

Can the exemption of the other patients be justly attributed to the cauterization of the wounds, and the amputation of the injured fingers? With respect to the case communicated to the Academy of Sciences in 1784, I conceive little doubt can remain in regard to the efficacy of the former of these means of prevention; for though this patient was bitten in twenty-five different parts, and though most of the wounds were of considerable extent, and inflicted on the naked parts of the body, yet no disease ensued.

Neither could any doubt be entertained of the madness of the dog in this instance, as another patient, who had the misfortune to be injured in the face, by the same animal, died hydrophobic. The other five cases afford only presumptive evidence, but of a nature so strong, that it is impossible to withhold our assent, as to the efficacy of the prophylactic means which were employed.

